# Supplementary material for: Risk of 28-day readmissions among stroke patients in Malaysia (2008–2015): Trends, causes and its associated factors
Source: PLoS One. 2021 Jan 19;16(1):e0245448. doi: 10.1371/journal.pone.0245448 (PMC7815148; doi:10.1371/journal.pone.0245448)
Supplement: Attachment — Submitted filename: Response to Reviewers.docx [file pone.0245448.s001.docx]

Dear Editor and Reviewer,

We thank you for the detailed review and constructive comments. Please find our response to your comments as listed below in the tables.

**Responses to comments from Reviewer #1**

| **Comments** | **Author’s Response** |
| --- | --- |
| 1. It would be interesting to know causes of readmission for every stroke subtype and factors associated; the rates of causes could be different according to subtype, and could have implications in strategies and measures to improve it. | Thank you for the comment. We have added causes of readmission by stroke subtypes in the Result section:  **Result, line 194-200, page 11**  When broken down by subtypes, the main causes were recurrent stroke (29.4%), pneumonia (14.1%) and sepsis (5.1%) among ischemic stroke patients; recurrent stroke (43.6%), pneumonia (9.8%) and injuries to the head (5.0%) among hemorrhagic stroke patients; and recurrent stroke (38.2%), disease of arteries, arterioles and capillaries (6.7%), and other cerebrovascular disease or sequelae (4.5%) among SAH patients. Overall, recurrent stroke remained the most common cause of readmission across all subtypes. (Fig 4) |
| 2. Could the author explore the role of stroke severity? I don’t know if scales as NIHSS were collected or registered. The work could gain relevance with this variable playing. If is not possible to get it, should be declared as a limitation. As the same way, ischemic stroke subtype (by TOAST or others classifications) could add more relevance to the study, knowing that prior recent studies have shown a decreased in stroke recurrence over the years. | Thank you for the suggestion. We agree that stroke severity as well as ischemic subtypes play an important role in determining readmissions of the stroke patients. However, the data used for this study came from an administrative database which did not collect the above information. We have included this as a limitation of our study. It now reads:  **Discussion, line 336-338, page 17**  Our study was limited by the lack of objective stroke-specific severity measurements, ischemic subtypes and other important clinical data such as comorbidities, functional status and prescription of preventive medication as the data were unavailable in this administrative database. |
| 3. A deeper discussion about the findings comparing with prior studies in other regions, mostly, in early readmissions causes. Please, the references should be verified. | Thank you for the comment. We have added few discussion points on causes of readmissions and rephrase part of the discussion. The references are verified. It now reads:  **Discussion, line 272-281, page 14-15**  The stroke-induced immunosuppression renders the patients more susceptible to infections after a stroke event. [19] In addition, common sequelae of stroke such as bladder dysfunction and dysphagia (with subsequent aspiration) predispose stroke patients to urinary tract infections and pneumonia. Quality of care after hospital discharge may also play a role. However, it may be arguable how many of these infections are actually preventable. On the other hand, the high frequency of readmissions due to recurrent stroke could reflect natural disease progression or a gap in secondary prevention of stroke. Prescriptions of antihypertensives drugs and oral anticoagulants among patients with ischemic stroke upon hospital discharge in Malaysia have been found to be suboptimal (48% and 33% respectively).[20] |

**Responses to comments from editor (****Dr Miguel A. Barboza)**

| 1. Please ensure that your manuscript meets PLOS ONE's style requirements, including those for file naming. | Thank you for the comment. We have made the necessary changes. |
| --- | --- |
| 2. You indicated that you had ethical approval for your study. In your Methods section, please ensure you have also stated whether you obtained consent from participants included in the study or whether the research ethics committee or IRB specifically waived the need for their consent. | We obtained waiver of informed consent from the ethics committee. The information is updated in the Methods section. It now reads:  **Methods, line 107-108, page 5**  The study was approved by the Medical Research Ethics Committee, Ministry of Health Malaysia (NMRR-18-100-39847) with waiver of informed consent. |
| 3. We noted in your submission details that a portion of your manuscript may have been presented or published elsewhere.   "The manuscript was presented in form of a poster and published in form of an abstract during the 6th Asia Pacific Conference on Public Health held on 22 – 25th July 2019 in Penang, Malaysia."  Please clarify whether this publication was peer-reviewed and formally published. If this work was previously peer-reviewed and published, in the cover letter please provide the reason that this work does not constitute dual publication and should be included in the current manuscript. | Thank you for the question. It was a poster presentation for a conference. The abstract for the poster was included in the conference proceeding and was not peer-reviewed. |
| 4. We note that you have indicated that data from this study are available upon request. PLOS only allows data to be available upon request if there are legal or ethical restrictions on sharing data publicly.   In your revised cover letter, please address the following prompts:  a) If there are ethical or legal restrictions on sharing a de-identified data set, please explain them in detail (e.g., data contain potentially identifying or sensitive patient information) and who has imposed them (e.g., an ethics committee). Please also provide contact information for a data access committee, ethics committee, or other institutional body to which data requests may be sent.  b) If there are no restrictions, please upload the minimal anonymized data set necessary to replicate your study findings as either Supporting Information files or to a stable, public repository and provide us with the relevant URLs, DOIs, or accession numbers. | We understand the concern and express our limitation in the cover letter:  All relevant data are presented within the manuscript. Study data are available from Health Informatics Centre, Ministry of Health Malaysia. Individual data cannot be made publicly available due to ethical and legal restrictions by the Personal Data Protection Act in Malaysia. Request for aggregate data may be sent to the Institute for Clinical Research at [contact@crc.gov.my](mailto:contact@crc.gov.my). |
